# Disability and sex/gender intersections in unmet workplace support needs: Findings from a large Canadian survey of workers

**DOI:** 10.1002/ajim.23203

**Published:** 2020-11-24

**Authors:** Arif Jetha, Monique A. M. Gignac, Selahadin Ibrahim, Kathleen A. Martin Ginis

**Affiliations:** ^1^ Institute for Work & Health Toronto Ontario Canada; ^2^ Dalla Lana School of Public Health University of Toronto Toronto Ontario Canada; ^3^ Krembil Research Institute Toronto Ontario Canada; ^4^ Department of Medicine, Division of Physical Medicine & Rehabilitation University of British Columbia Vancouver British Columbia Canada; ^5^ School of Health and Exercise Sciences University of British Columbia Kelowna British Columbia Canada; ^6^ Centre for Chronic Disease Prevention and Management, Southern Medical Program University of British Columbia Kelowna Canada

**Keywords:** disability, intersectionality, job accommodations, sex/gender differences, work conditions

## Abstract

**Introduction:**

Individual attributes including disability and sex/gender have the potential to intersect and determine the likelihood of unmet workplace support needs. Our study compares unmet workplace support needs between workers with and without a disability, and according to disability type and sex/gender differences.

**Methods:**

Workers with (*n* = 901) and without (*n* = 895) a disability were surveyed to examine their need and use of workplace supports including job accommodations, work modifications and health benefits. A multivariable logistic model was conducted to examine the relationship between disability status, disability type and sex/gender and unmet workplace support needs. The model included interaction terms between sex/gender × physical disability, sex/gender × nonphysical disability, and sex/gender × physical and nonphysical disability.

**Results:**

Among participants with a disability, 24% had a physical disability, 20% had a nonphysical disability (e.g., cognitive, mental/emotional or sensory disability) and 56% had both physical and nonphysical disability. Over half of the respondents were women (56%). Results from the multivariable model showed that nondisabled women were more likely to report unmet workplace support needs when compared to nondisabled men (odds ratio [OR] = 1.54, 95% confidence interval [CI], 1.13–2.10). Findings also showed an intersection between the number and type of disability and sex/gender; women with both a physical and nonphysical disability had the greatest likelihood of reporting unmet workplace support needs when compared to nondisabled men (OR = 2.73; 95% CI, 1.83–4.08).

**Conclusions:**

Being a woman and having one or more disabilities can determine unmet workplace support needs. Strategies to address workplace support needs should consider the intersection between disability and sex/gender differences.

## 1 BACKGROUND

Within the workplace, the supports an employee receives play an important role in addressing the presence of barriers to productive employment. Individual attributes including whether a person has a disability and their sex/gender can determine their experiences in the labor market and the extent to which workplace supports are needed and used. Individual attributes have the potential to intersect and could exacerbate the likelihood of unmet workplace support needs. In a large sample of Canadian workers, our study examines the intersection between disability and sex/gender as they relate to whether workplace support needs are met. Findings will bring to light subgroups who are more likely to face inequities in working conditions. Also, our study will generate insights that can inform practical solutions to support employment for people with and without disabilities.

A body of research indicates that people living with physical and nonphysical disabilities (e.g., mental health, cognitive and sensory disabilities) face challenges in finding and sustaining productive employment compared to their counterparts without a disability.^1^, ^2^, ^3^, ^4^, ^5^ Workplace supports modify aspects of the work context that can contribute to employment limitations.^6^ Studies indicate that the most‐needed workplace supports reported by people with disabilities include health benefits (e.g., prescription drug coverage), work modification (e.g., scheduling flexibility), and job accommodation (e.g., accessible workstations).^6^, ^7^, ^8^, ^9^, ^10^, ^11^ People with disabilities also indicate that workplace support needs are often unmet. Within industrialized contexts, studies point to a lack of employer awareness regarding the legal requirement for reasonable accommodations or an understanding of how to adjust the work environment for people with different disabling conditions.^5^, ^6^, ^12^ Unmet workplace support needs can also be attributed to difficulties faced by people with disabilities in communicating their requirements at work, reporting a lack of control over their working conditions, apprehension to receive special treatment in comparison to their nondisabled colleagues, or fear of losing career development opportunities.^8^, ^9^ Of significance, research also indicates that workplace supports can be beneficial to a range of workers including those who face limitations attributed to child and elder care responsibilities, aging, or pregnancy.^13^, ^14^, ^15^, ^16^, ^17^ Accordingly, workers without a disabling health condition may also benefit from similar workplace supports to those required by people with disabilities. Currently, limited research exists which compares the extent to which workers with and without disabilities differ in terms of their need and use of workplace supports.

It is important to highlight that working experiences can differ according to a range of intersecting disability and personal factors.^2^ Intersectionality offers a lens to interpret the combined effects of individual identities that cannot be disaggregated or understood separately.^18^, ^19^ The application of intersectionality has commonly utilized qualitative methods to examine how people at the nexus of different social identities experience multiple and overlapping challenges in different social domains. Intersectionality underscores one's experiences, context, and intersecting power structures that emerge from macrolevel forces and contribute to advantage or disadvantage within the working world.^20^, ^21^, ^22^, ^23^ In studies of people with disabilities, intersectionality has provided deeper insight into the experiences of a heterogeneous population including subgroups who face greater disadvantage or privilege in the labor market.^24^, ^25^ In this study we draw from an intersectional framework to examine how disability factors and sex/gender differences can interact to influence unmet workplace support needs.

The number and type of disabilities can determine the likelihood of finding employment, and sustaining productivity.^26^, ^27^, ^28^ Some research indicates that nonphysical disabilities (i.e., mental health or cognitive impairments) are associated with significant work functioning restrictions when compared to physical disabilities.^29^ These same studies indicate that workers with both a physical and nonphysical disability are more likely to experience challenges within the labor market when compared to those living with either a physical or nonphysical disability alone.^28^, ^29^ Few studies have examined how workplace support need and use differs for those reporting different disability types and numbers.

Unmet workplace support needs also has the potential to differ according to sex/gender. Studies highlight differences between women and men based on sex (biology) and gender (sociocultural) in occupation and educational attainment, need for job accommodation, availability of career advancement opportunities, remuneration, productivity, job control, exposure to workplace hazards, and work‐life balance.^30^, ^31^, ^32^, ^33^, ^34^, ^35^, ^36^ Recent population‐level data indicate that disability prevalence is greater among women than men.^3^ Men with disabilities more frequently hold paid employment, and are more likely to work full‐time hours and earn a higher income when compared to women with disabilities.^3^ Growing methodological literature has examined sex and gender measures in work and health research.^37^ In the absence of specific measures, taking a more inclusive approach to assessing biological and sociocultural determinants can enable researchers to examine sex/gender differences.^38^ For instance, using an open‐ended self‐report measure that captured dimensions of sex and gender, a recent cross‐sectional survey of 463 older men (*n* = 197) and women (*n* = 266) with arthritis investigated the availability and need of 14 workplace supports.^38^ When compared to participants who were men, women participants more frequently reported needing >5 workplace supports (21% vs. 35%) and having unmet workplace support needs (20% vs. 27%).^38^ To elaborate on existing evidence, research of a broader age‐range of working adults with and without disabilities is required to compare unmet workplace support needs for women and men.^38^

## 2 AIMS AND HYPOTHESES

To date, limited studies of people with disabilities have leveraged quantitative survey data to examine how sex/gender and disability can intersect and influence labor market experiences.^18^, ^25^, ^39^ We utilize data from a large community‐based survey to compare the likelihood of reporting unmet workplace support needs between people with and without disability. Our study also aims to examine the intersection between disability status and type, and sex/gender and the likelihood of unmet workplace support needs. We hypothesize that:

1. Participants reporting a disability will be more likely to report unmet workplace support needs when compared to their counterparts without a disability.
2. Participants who report living with both physical and nonphysical disability will be more likely to report unmet workplace support needs when compared to those who report living with either a physical or nonphysical disability.
3. Sex/gender will intersect the relationship between disability and unmet workplace support needs, such that the relationship between disability and unmet workplace support needs will be most pronounced for women when compared to men.
4. The intersection between sex/gender and disability and its relationship with unmet workplace support needs will be additive, such that unmet workplace support needs will be most pronounced in women living with both a physical and nonphysical disability when compared to men not living with a disability.

Results have the potential to contribute to recommendations for the design and delivery of policies and programs that promote greater inclusion for people living with disabilities and other workers within the labor market.^40^

## 3 METHODS

To test the study hypotheses, an online survey of Canadians with and without disabilities was conducted from July to August 2018. Eligibility criteria included being ≥18 years of age and fluent in English. To capture working experiences, eligible participants had to hold paid employment for at least ≥15  h/week. Given the challenge associated with recruiting large community‐based samples of people with disabilities necessary to test interaction effects in multivariable models,^41^ survey participants were identified from an existing panel that is maintained by a research firm. The panel consists of over one million Canadians and is nationally representative according to region and income.^42^ Using demographic information held by the research firm, a purposive sampling strategy was utilized to identify potential panel members that met eligibility criteria. Potential participants identified from the panel were contacted and provided information about the study and asked to complete a short screening questionnaire to verify study eligibility. For those who chose to participate, informed consent was obtained, and the online survey was administered. Of the potential participants who viewed the study invitation, met eligibility criteria, and provided consent, 88% completed the survey. To ensure confidentiality, participants were assigned an anonymous identification number, data were stored on a secure server accessible only to the research team, and data were presented in the aggregate form. Study procedures were reviewed by the University of Toronto's research ethics board (REB# 36184).

### 3.1 Measures

#### 3.1.1 Outcome: Unmet workplace support needs

A list of 12 workplace supports including job accommodations (e.g., assistive devices, accessible workplace), work modifications (e.g., scheduling flexibility, modified job duties) and health benefits (e.g., prescription drug coverage, employee assistance program) were presented to participants. Items were drawn from previously published qualitative and quantitative studies of people living with disabilities.^9^, ^11^ Unmet workplace support needs were determined by asking participants about their needs and the use of each workplace support. First, participants were asked: “Regardless of whether they were available to you, which of the following benefits, policies or workplace accommodations have you needed in the past year?” (1 = needed, 0 = not needed). Second, participants were asked about their use of each workplace supports using the question: “Regardless of whether they were available to you or whether you needed them, which of the following benefits, policies or workplace accommodations have you used in the past year and how often have you used them?” (1 = used, 0 = not used). A participant was categorized as having unmet workplace support needs when the total number of needed workplace supports exceeded the number of workplace supports used.^10^ Notably, our approach enabled us to capture the need and usage of workplace supports that could be formally provided by an employer or infomally accessed by a worker.^10^

#### 3.1.2 Predictor variables

##### 3.1.2.1 Disability

Participants were asked about difficulties related to four categories that included physical, cognitive, mental/emotional, or sensory disability.^43^ Item response occurred on a four‐point scale (0 = no, 1 = sometimes, 2 = often, 3 = always). Participants who reported having sometimes difficulty on at least one item were categorized as having a disability.^43^

##### 3.1.2.2 Sex/gender

Participants were asked an open‐ended question to identify their sex/gender. Participants were then categorized as either women, men or nonbinary. We label this variable as sex/gender to account for biological and social dimensions that will be captured by self‐report.^38^

#### 3.1.3 Covariates

Sociodemographic factors, health factors, and work characteristics were included in our analysis as covariates and selected according to their relationship with disability and work identified in previous research.^1^

##### 3.1.3.1 Sociodemographic

Information on age (years), educational attainment (1 = primary to high school, 2 = some postsecondary, 3 = graduated postsecondary), marital status, and personal income was collected.

##### 3.1.3.2 Health factors

Self‐rated health was assessed (1 = poor health, 5 = excellent health). Also, self‐reported pain and fatigue were examined using visual analog scales (0 = no pain/fatigue, 10 = worst possible pain/fatigue).^44^

##### 3.1.3.3 Work characteristics

Information on job sector (business/administration/technology, health/science/teaching, sales/service, and trades/transportation sectors), job tenure (years), hours worked/week and organizational size were collected (e.g., small [1–50 people], medium [51–150 people], and large [>150 people]). Two questions asked about the extent to which a participant's job was physically and mentally demanding (1 = not at all, 5 = a great deal). Participants were also asked about perceptions of job control (1 = not at all, 5 = a great deal) and job stress (1 = not at all, 5 = a great deal). The eight‐item version of the Work Limitations Questionnaire was utilized to measure limitations on job performance and productivity related to a disability.^45^ Limitations were assessed on five dimensions (e.g., time management, physical demands, mental/interpersonal demands, and output) and item response occurred on a four‐point scale (0 = none of the time (0%), 4 = all of the time (100%)). Items from each dimension were weighted and summed to produce a total score ranging from 0.4 to 28.6, with a higher score reflecting fewer overall limitations.^45^ Lastly, absenteeism was assessed by asking participants about the number of missed work days due to health in the past 3 months.

#### 3.1.4 Analyses

Descriptive statistics (i.e., frequencies, percentages, means, and standard deviation (*SDs*)) built a profile of respondents to examine the distribution of study variables. To sustain statistical power for subsequent analytical approaches, participants with nonphysical disabilities were grouped together (e.g., mental/emotional, cognitive and sensory disabilities). *χ^2^* test and analysis of variance were conducted to examine if study variables differed across four groups including: no disability, physical disability, nonphysical disability and both physical and nonphysical disability.

Univariable logistic models were developed to examine the association between predictor variables, study covariates, and unmet workplace support needs. Separate univariable logistic regression models were conducted for the total sample, those not living with a disability, and those living with a physical, nonphysical disability or both a physical and nonphysical disability. Covariates significantly associated with unmet workplace support needs in at least one group were carried forward to the final multivariable model. Also, variables of theoretical importance in the relationship between disability and unmet workplace support needs or have been associated with sex/gender differences in employment experiences were carried forward. A multivariable logistic regression model was conducted which included sex/gender and physical disability, nonphysical disability, and both physical and nonphysical disability as study covariates. The multivariable model also included the interaction terms between sex/gender × physical disability, sex/gender × nonphysical disability, and sex/gender × physical and nonphysical disability. To enable the comparison of primary independent variables and their interaction effects, the reference category in the multivariable model was set to men not reporting a disability. Model fit was determined using the Hosmer‐Lemeshow goodness‐of‐fit test.

## 4 RESULTS

A total of 1796 employed participants with (*n* = 895) and without disabilities (*n* = 901) completed the survey and had minimal missing data. A description of the study participants is provided in Table 1. Among those living with a disability, 24% had a physical disability, 20% indicated a nonphysical disability, and 56% reported both physical and nonphysical disability. Just over half of the sample were women (56%) and no participants in the study sample identified as nonbinary. A greater proportion of participants with a nonphysical disability (67%) and physical and nonphysical disability (57%) were women when compared to those reporting a physical disability (52%) or no disability (55%). Over half of the full sample were married/living as if married (57%). An examination of health factors indicated a mean pain score of 2.6 (*SD* = 2.7) and a mean fatigue score of 3.7 (*SD* = 2.9) for the full sample. Also, in the full sample, participants indicated mean self‐rated health of 3.0 (*SD* = 1.0). Those living with both a physical and nonphysical disability reported greater mean pain (mean = 4.5, *SD* = 2.7) and fatigue (mean = 5.7, *SD* = 2.6) and lower mean self‐rated health (mean = 2.4, *SD* = 0.9) when compared to participants without a disability or those reporting either a physical or nonphysical disability.

**Table 1:** Sample characteristics for the full sample as well as for participants with no disability and those reporting a physical, nonphysical, and physical and nonphysical disability

	**Full sample (*n* = 1796)**	**No disability (*n* = 901)**	**Physical disability (*n* = 216)**	**Nonphysical disability (*n* = 172)**	**Physical and nonphysical disability (*n* = 507)**	
	**Mean ± *SD*/*N* (%)**	**Mean ± *SD*/*N* (%)**	**Mean ± *SD*/*N* (%)**	**Mean ± *SD*/*N* (%)**	**Mean ± *SD*/*N* (%)**	***p* value** **
*Sociodemographic*						
Sex/gender (women)	1011 (56.3)	494 (54.8)	112 (51.9)	116 (67.4)	289 (57.1)	.0100
Age (years)						<.0001
18–35	597 (33.2)	301 (33.4)	29 (13.4)	88 (51.2)	179 (35.3)	
36–50	600 (33.4)	300 (33.3)	71 (32.9)	60 (34.9)	169 (33.3)	
>50	599 (33.4)	300 (33.3)	116 (53.7)	24 (13.9)	159 (31.4)	
Marital status						.0001
Married/living as married	1018 (56.8)	529 (58.9)	129 (59.7)	83 (48.5)	277 (54.7)	
Widowed/divorced/separated	217 (12.1)	94 (10.5)	39 (18.1)	15 (8.8)	69 (13.6)	
Never married	556 (31.0)	275 (30.6)	48 (22.2)	73 (42.7)	160 (31.6)	
Education level						.0001
Primary to high school	334 (18.6)	142 (15.8)	51 (23.7)	28 (16.3)	113 (22.3)	
Some postsecondary	148 (27.5)	222 (24.7)	64 (29.8)	58 (33.7)	148 (29.3)	
Graduated postsecondary	965 (53.9)	534 (59.5)	100 (46.5)	86 (50.0)	245 (48.4)	
Income						.0007
<$50,000	607 (35.8)	260 (30.7)	80 (39.8)	68 (41.5)	199 (41.1)	
$50,000–$89,999	624 (36.8)	321 (37.9)	70 (34.8)	61 (37.2)	172 (35.5)	
≤$90,000	465 (27.4)	266 (15.7)	51 (25.4)	35 (21.3)	113 (23.4)	
*Work context*						
Job sector						.3097
Business/administration/technology	419 (23.4)	220 (24.5)	51 (23.8)	38 (22.2)	110 (21.8)	
Health/science/teaching	609 (34.1)	314 (34.9)	66 (30.8)	65 (38.0)	164 (32.5)	
Sales/service	392 (21.9)	197 (21.9)	41 (19.2)	33 (19.3)	121 (23.9)	
Trades/transportation	369 (20.6)	168 (18.7)	56 (26.2)	35 (20.5)	110 (21.8)	
Organization size						.0015
Small (1–50)	437 (25.5)	224 (25.8)	54 (26.3)	49 (30.2)	110 (23.0)	
Medium (51–150)	318 (18.5)	134 (15.4)	36 (17.6)	28 (17.3)	120 (25.0)	
Large (>150)	960 (56.0)	511 (58.8)	115 (56.1)	85 (52.5)	249 (52.0)	
Job tenure (years)	9.5 ± 9.0	9.7 ± 9.0	12.3 ± 10.7	6.9 ± 7.5	9.0 ± 8.3	<.0001
Work hours (h/week)	37.8 ± 8.9	38.2 ± 8.8	38.4 ± 10.3	37.1 ± 8.4	37.2 ± 8.5	.1382
Perceived job stress (1–5)	2.9 ± 1.0	2.6 ± 1.0	2.9 ± 1.0	3.0 ± 0.9	3.3 ± 1.0	<.0001
Perceived job control (1–5)	2.8 ± 1.2	2.9 ± 1.2	2.7 ± 1.3	2.7 ± 1.3	2.7 ± 1.2	.0005
Physical job demands (1–5)	2.9 ± 1.4	2.7 ± 1.4	3.1 ± 1.5	2.8 ± 1.5	3.2 ± 1.3	<.0001
Mental job demands (1–5)	3.6 ± 1.2	3.4 ± 1.2	3.6 ± 1.2	3.7 ± 1.2	3.8 ± 1.1	<.0001
Productivity loss (WLQ: 0–28.6)	4.6 ± 5.5	1.9 ± 3.6	4.4 ± 4.6	5.6 ± 5.2	8.8 ± 5.4	<.0001
Absenteeism in previous 3 months (0–90)	4.3 ± 12.9	1.9 ± 8.6	5.0 ± 15.2	3.0 ± 8.6	8.6 ± 17.7	<.0001
*Health*						
Pain (0–10)	2.6 ± 2.7	1.4 ± 1.9	3.5 ± 2.7	2.5 ± 2.5	4.5 ± 2.7	<.0001
Fatigue (0–10)	3.7 ± 2.9	2.3 ± 2.4	4.2 ± 2.7	4.5 ± 2.9	5.7 ± 2.6	<.0001
Self‐rated health (1–5)	3.0 ± 1.0	3.5 ± 0.9	2.8 ± 1.0	2.8 ± 0.9	2.4 ± 0.9	<.0001
Workplace support needs						
Workplace support needs met	978 (54.5)	555 (60.5)	111 (51.4)	81 (47.1)	241 (47.6)	<.0001
Workplace support needs unmet	817 (45.5)	356 (39.5)	105 (48.6)	91 (52.9)	265 (52.4)	

*Note*: Sample sizes vary due to different numbers of missing values for different variables.

Abbreviation: WLQ, work limitations questionnaire.

** To test differences across each group used *χ* ^2^ tests were used for categorical variables and analysis of variance was used for continuous variables.

A description of work context factors is provided in Table 1. Respondents indicated mean job tenure of 9.5 years (*SD* = 9.0) and mean work hours of 37.8/week (*SD* = 8.9). Over one‐third of participants worked in health/science/teaching job sectors (34%) and over half (56%) worked in large organizations. Participants reported mean perceived job stress and control of 2.9 (*SD* = 1.0) and 2.8 (*SD* = 1.2), respectively. Also, participants reported mean physical and mental job demands of 2.9 (*SD* = 1.4) and 3.6 (*SD* = 1.2), respectively. Those living with both a physical and nonphysical disability reported significantly greater job stress (mean = 3.3, *SD* = 1.0) and greater physical (mean = 3.2, *SD* = 1.3) and mental job demands (mean = 3.8, *SD* = 1.1) when compared to those not reporting a disability or either a physical or nonphysical disability. Participants indicated a mean productivity loss score of 4.6 (*SD* = 5.5) and a mean of 4.3 days absent attributed to their health in the last 3 months (*SD* = 12.9). Participants with both a physical and nonphysical disability reported significantly greater productivity loss (mean = 8.8, *SD* = 5.4) and work days absent (mean = 8.6, *SD* = 17.7) when compared to participants with no disability or those with a physical or nonphysical disability (Table 1).

**Figure 1:**
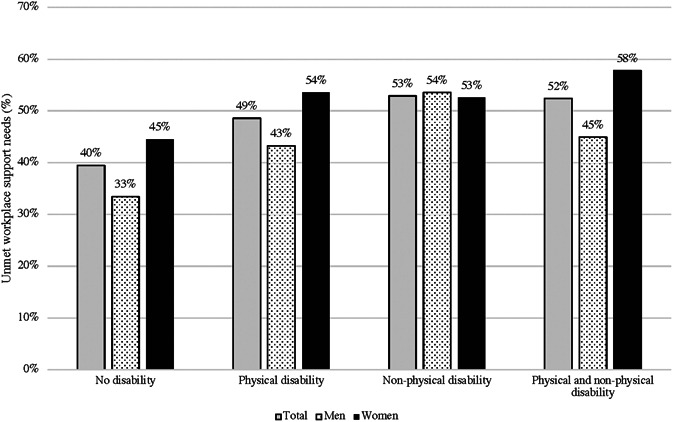
Frequency of unmet workplace support needs reported for male and female participants. Findings are presented for the total sample, participants with no disability, as well as those reporting physical, nonphysical, and physical and nonphysical health disability

Just under half of the participants reported that their workplace support needs were unmet (46%). Participants with a physical (49%), nonphysical disability (53%) or both physical and nonphysical disability (52.4%) were more likely to report unmet workplace support needs when compared to participants without a disability (39.5%; *p* < .0001; Table 1 and Figure 1).

Across the full sample, the most frequently needed workplace supports included prescription drug coverage (76%), scheduling flexibility (64%), extended health benefits (61%), modified job duties (41%), and informal job modification (40%). Across all workplace supports, participants living with both a physical and nonphysical disability most frequently reported greater needs when compared to those not reporting a disability or those reporting either a physical or nonphysical disability. The most utilized workplace supports were prescription drug coverage (70%), scheduling flexibility (50%), and extended health benefits (50%). Across all workplace supports, participants living with both physical and nonphysical disability most often reported greater use when compared to those reporting either a physical or nonphysical disability or those not reporting a disability (Table 2).

**Table 2:** Needed and used workplace supports for the full sample, as well as for participants not living with a disability and for those with a physical disability, nonphysical disability, and physical and nonphysical disability

	Need						Used					
	Full sample (%)	No disability (%)	Physical disability (%)	Nonphysical disability (%)	Physical and nonphysical disability (%)	*p* value	Full sample (%)	No disability (%)	Physical disability (%)	Nonphysical disability (%)	Physical and nonphysical disability (%)	*p* value**
Prescription drug coverage	76.2	72.7	76.4	78.5	81.7	.002	69.5	67.3	69.3	69.2	73.7	.10
Scheduling flexibility	64.2	56.4	62.5	74.4	75.4	<.0001	50.0	41.5	49.1	56.1	63.4	<.0001
Extended health benefits	60.8	54.0	57.9	62.2	73.9	<.0001	49.9	45.6	49.3	45.6	59.2	<.0001
Modified job duties	40.5	27.8	38.9	46.8	61.7	<.0001	29.4	18.1	29.3	32.6	48.4	<.0001
Informal job modification	40.1	27.2	45.8	42.1	60.2	<.0001	28.3	18.0	29.9	26.7	46.6	<.0001
Workstation modifications	38.5	28.7	36.3	38.9	56.6	<.0001	26.8	18.2	26.2	25.7	42.7	<.0001
Accessible workplace	38.2	27.7	38.0	39.0	56.8	<.0001	28.0	19.2	27.0	23.8	45.6	<.0001
Facilities to manage health	37.7	25.9	36.6	42.7	57.2	<.0001	26.3	17.8	22.7	27.5	42.7	<.0001
Employee assistance program	36.5	24.4	33.5	42.1	57.5	<.0001	26.4	16.2	25.9	29.7	43.8	<.0001
Work‐from‐home arrangements	35.9	31.9	27.6	42.3	44.4	<.0001	27.9	25.1	21.0	28.4	35.7	<.0001
Communication adaptation	30.3	21.7	29.8	32.6	45.3	<.0001	21.1	14.8	17.7	18.8	34.7	<.0001
Assistive devices	28.9	21.7	25.9	29.7	42.7	<.0001	19.8	14.7	18.6	15.3	31.0	<.0001

** To test differences across each group used*χ* ^2^ tests were utilized.

Frequency of unmet workplace support needs is presented in Figure 1 according to sex/gender differences (Figure 1). Women more frequently reported unmet workplace support needs when compared to male respondents not reporting a disability (45% vs. 33%). Women with a physical disability were more likely to report unmet workplace support needs when compared to men with a physical disability (54% vs. 43%). Additionally, women with both physical and nonphysical disability were more likely to report unmet workplace support needs when compared to their men with both a physical and nonphysical disability (58% vs. 45%) (Table 2).

At the univariable level (Table 3), participants reporting a disability including a physical disability (odds ratio [OR] = 1.44; 95% confidence interval [CI], 1.08–1.95), nonphysical disability (OR = 1.72; 95% CI, 1.24–2.39) and both physical and nonphysical disability (OR = 1.68; 95% CI, 1.35–2.10) were significantly more likely to report unmet workplace support needs when compared to those not reporting a disability. At the univariable level, being a woman was associated with a greater likelihood of unmet workplace support needs when compared to men (OR = 1.56; 95% CI, 1.29–1.89). At the univariable level, the relationship between being a woman and greater unmet workplace support needs was significant for those not reporting a disability (OR = 1.60; 95% CI, 1.22–2.10), as well as those reporting both a physical and nonphysical disability (OR = 1.68; 95% CI, 1.18–2.40).

**Table 3:** Univariable logistic regression examining the factors associated with unmet workplace support needs for the full sample, as well as for participants with no disability and those reporting a physical, nonphysical, or physical and nonphysical disability

	**Full sample**	**No disability**	**Physical disability**	**Nonphysical disability**	**Physical and nonphysical disability**
	**OR (95% CI)**	**OR (95% CI)**	**OR (95% CI)**	**OR (95% CI)**	**OR (95% CI)**
*Disability status*					
No disability	ref	–	–	–	–
Physical disability	**1.44 (1.07, 1.95)**				
Nonphysical disability	**1.72 (1.24, 2.39)**				
Physical and nonphysical disability	**1.68 (1.35, 2.10)**				
*Sociodemographic*					
Sex/gender (women)	**1.56 (1.29, 1.89)**	**1.60 (1.22, 2.10)**	1.51 (0.88, 2.59)	0.96 (0.51, 1.82)	**1.68 (1.18, 2.40)**
Age (years)					
18–35	ref	ref	ref	ref	ref
36–50	**0.76 (0.61, 0.96)**	**0.57 (0.41, 0.79)**	0.88 (0.37, 2.09)	0.89 (0.46, 1.72)	1.14 (0.75, 1.73)
>50	0.91 (0.73, 1.14)	**0.71 (0.51, 0.98)**	1.11 (0.49, 2.50)	0.83 (0.34, 2.06)	1.36 (0.89, 2.10)
Marital status					
Married/living as married	ref	ref	ref	ref	ref
Widowed/divorced/separated	1.01 (0.82, 1.25)	1.00 (0.74, 1.36)	0.66 (0.34, 1.29)	0.64 (0.34, 1.20)	1.35 (0.92, 2.00)
Never married	1.22 (0.91, 1.64)	1.21 (0.78, 1.89)	0.79 (0.39, 1.63)	1.09 (0.36, 3.36)	1.45 (0.85, 2.47)
Education level					
Primary to high school	1.12 (0.88, 1.44)	0.87 (0.81, 1.63)	0.92 (0.47, 1.81)	0.47 (0.20, 1.11)	**1.81 (1.15, 2.85)**
Some postsecondary	1.03 (0.83, 1.28)	1.11 (0.81, 1.53)	0.61 (0.32, 1.15)	0.51 (0.26, 0.99)	1.27 (0.85, 1.92)
Graduated postsecondary	ref	ref	ref	ref	ref
Income					
<$50,000	ref	ref	ref	ref	ref
$50,000–$89,999	0.82 (0.65, 1.02)	0.72 (0.52, 1.00)	1.08 (0.57, 2.06)	1.43 (0.71, 2.86)	0.81 (0.54, 1.22)
≤$90,000	**0.70 (0.55, 0.90)**	**0.69 (0.49, 0.98)**	1.24 (0.61, 2.50)	1.26 (0.56, 2.85)	**0.58 (0.36, 0.92)**
*Work context*					
Job sector					
Business/administration/technology	0.86 (0.65, 1.14)	1.00 (0.66, 1.51)	0.87 (0.40, 1.87)	1.62 (0.64, 4.11)	0.59 (0.34, 1.00)
Health/science/teaching	1.18 (0.91, 1.53)	1.34 (0.91, 1.97)	1.58 (0.77, 3.24)	1.69 (0.74, 3.89)	0.80 (0.49, 1.30)
Sales/service	1.06 (0.79, 1.41)	1.01 (0.66, 1.54)	1.30 (0.58, 2.92)	0.53 (0.20, 1.41)	1.38 (0.81, 2.34)
Trades/transportation	ref	ref	ref	ref	ref
Organization size					
Small (1–50)	ref	ref	ref	ref	ref
Medium (51–150)	**0.72 (0.54, 0.96)**	**0.64 (0.41, 0.99)**	0.62 (0.26, 1.44)	1.11 (0.44, 2.81)	0.66 (0.39, 1.11)
Large (>150)	**0.78 (0.62, 0.98)**	0.77 (0.56, 1.06)	0.85 (0.44, 1.62)	1.19 (0.59, 2.40)	0.69 (0.44, 1.08)
Job tenure (years)	0.99 (0.98,1.00)	0.99 (0.97, 1.01)	1.00 (0.97, 1.02)	0.99 (0.95, 1.03)	0.99 (0.97, 1.01)
Work hours (h/week)	**0.99 (0.98, 0.99)**	0.99 (0.97, 1.00)	0.99 (0.97, 1.02)	1.00 (0.97, 1.04)	**0.98 (0.98, 0.99)**
Perceived job stress (1–5)	**1.16 (1.05, 1.27)**	1.13 (0.98, 1.30)	0.97 (0.74, 1.27)	1.09 (0.79, 1.50)	1.08 (0.90, 1.29)
Perceived job control (1–5)	1.00 (0.93, 1.08)	1.03 (0.92, 1.15)	1.14 (0.93, 1.41)	0.98 (0.78, 1.25)	0.96 (0.82, 1.11)
Physical job demands (1–5)	1.00 (0.93, 1.06)	0.94 (0.86, 1.04)	0.95 (0.79, 1.14)	0.84 (0.68, 1.04)	1.08 (0.95, 1.24)
Mental job demands (1–5)	1.08 (1.00, 1.17)	1.01 (0.90, 1.13)	1.11 (0.89, 1.39)	0.99 (0.77, 1.28)	1.11 (0.95, 1.30)
Productivity loss (WLQ: 0–28.6)	1.00 (1.00, 1.02)	0.96 (0.92, 1.00)	0.99 (0.93, 1.05)	0.99 (0.93, 1.05)	0.99 (0.94, 1.00)
Absenteeism in past 3 months (0–90)	1.00 (1.00, 1.01)	1.01 (0.99, 1.02)	0.99 (0.97, 1.01)	0.98 (0.94, 1.02)	1.00 (0.99, 1.01)
*Health factors*					
Pain (0–10)	**1.04 (1.00, 1.07)**	1.02 (0.95, 1.09)	0.99 (0.90, 1.09)	1.06 (0.94, 1.20)	0.94 (0.88, 1.01)
Fatigue (0–10)	**1.05 (1.01, 1.08)**	1.00 (0.94, 1.06)	0.97 (0.87, 1.07)	1.04 (0.94, 1.16)	1.02 (0.96, 1.10)
Self‐rated health (1–5)	**0.81 (0.74, 0.89)**	**0.85 (0.74, 0.99)**	0.91 (0.69, 1.20)	0.83 (0.60, 1.14)	0.93 (0.76, 1.13)

*Note*: Sample sizes vary due to different numbers of missing values for different variables; bolded estimates represent those that are significantly related to unmet workplace support needs.

Abbreviations: CI, confidence interval; OR, odds ratio; ref, reference category; WLQ, work limitations questionnaire.

Our final multivariable model examined the interaction between sex/gender and disability status and the likelihood of reporting unmet workplace support needs to test study hypotheses (Table 4 and S1). The final model adjusted for sociodemographic, health and work context covariates and exhibited good model fit (Hosmer‐Lemeshow Goodness of fit test > .50). Findings indicated that when compared to men not living with a disability (reference category), men with a nonphysical disability (OR = 2.27; 95% CI, 1.24–4.15) and men with a physical and nonphysical disability (OR = 1.64; 95% CI, 1.10–2.50) were more likely to report unmet workplace support needs. Interestingly, when compared to men not living with a disability, women not living with a disability were more likely to report unmet workplace support needs (OR = 1.54; 95% CI, 1.13–2.10). Also, women participants with a physical disability (OR = 2.33; 95% CI, 1.44–3.78) and nonphysical disability (OR = 2.38; 95% CI, 1.46–3.88) were more likely to report unmet workplace support needs when compared to men without a disability. Women with both a physical and nonphysical disability had the greatest likelihood of reporting unmet workplace support needs when compared to men without a disability (OR = 2.73; 95% CI, 1.83–4.08).

**Table 4:** Multivariable logistic regression model of examining unmet workplace support needs when considering sex/gender and disability type (*n* = 1796)

	**OR**	**95% CI**
Men, no disability	ref	
Men, physical disability	1.57	0.97, 2.53
Men, nonphysical disability	**2.27**	**1.24, 4.15**
Men, physical and nonphysical disability	**1.64**	**1.09, 2.49**
Women, no disability	**1.54**	**1.13, 2.09**
Women, physical disability	**2.33**	**1.44, 3.78**
Women, nonphysical disability	**2.38**	**1.46, 3.88**
Women, physical and nonphysical disability	**2.73**	**1.83, 4.08**

*Note*: Models adjusted for age, marital status, education level, pain, fatigue, self‐rated health, productivity loss, work hours, job stress, job control, and absenteeism; bolded estimates represent those that are significantly related to unmet workplace support needs; Hosmer and Lemeshow goodness of fit test (*χ* ^2^ =  5.47(8), *p *= .71). We provide ORs for study covariates in Appendix A, Table A1.

Abbreviations: CI, confidence interval; OR, odds ratio; ref, reference category.

## 5 DISCUSSION

Workplace supports including health benefits, job accommodations, and work modifications play a critical role in addressing the limitations to employment participation and fostering labor market engagement. Utilizing a large a sample of employed Canadians, our study indicated that those with a disability were more likely to report unmet workplace support needs when compared to their counterparts without a disability. Notably, we examined the intersection between disability and sex/gender to determine those who may be most susceptible to unmet workplace support needs. Findings highlighted the challenges faced by women in the workplace when it comes to having workplace support needs met. Women living with both a physical and nondisability were more likely to report unmet workplace support needs when compared to men. Employer‐based policies and programs that encourage access to workplace support are required to meet the needs of people living with disabilities. Approaches to promoting the utilization of workplace supports that are tailored to women and those living with more than one disability could be beneficial to facilitate employee engagement.

Workplace supports represent an important employer‐led strategy to address the challenges that can impact sustained and productive employment.^12^ Aligning with previous research, participants in our study required diverse workplace supports to encourage labor market engagement including prescription drug coverage, scheduling flexibility, extended health benefits, modified job duties, and opportunities to informally modify work.^8^, ^9^, ^10^, ^11^ Interestingly, the types of workplace support that were most needed were similar for those with and without disabilities. A body of research highlights that workers report personal responsibilities (e.g., child and eldercare responsibility), health‐related demands (e.g., attending healthcare appointments), and workplace factors (e.g., interpersonal conflict) that can interrupt employment engagement.^13^, ^14^, ^15^, ^16^, ^17^, ^46^, ^47^, ^48^ Our findings have implications for employers who may be developing approaches to accommodate workers with different personal and health needs. The provision of similar types of workplace supports can be beneficial to meet the needs of a diverse range of employees including those with and without disabilities. Results may also point to the importance of universal design in accommodation planning within the workplace.^49^ By designing supports that are available and suitable for a diverse workforce, employers can increase accessibility and address a broader range of work limitations. A universal design approach can also address inequities that may exist with regard to the availability of workplace supports.

Supporting hypothesis one, participants with a disability were more likely to report unmet workplace support needs when compared to participants without a disability. Results align with previous research, and sheds more light on the challenges faced by workers with disabilities in accessing workplace support.^9^, ^11^ Over half of the participants reporting a disability in our study indicated both a physical and nonphysical disability. According to findings from the multivariable model, those with both physical and nonphysical disability experienced the greatest odds of unmet workplace support needs when compared to those reporting either a physical or nonphysical disability. Past research highlights an additive effect of living with more than one disability on the severity of work limitations.^27^, ^28^, ^29^, ^50^ For instance, population‐level data from Canada shows that the co‐occurrence of physical and mental health disability were significantly associated with role functional impairment and employment participation restrictions when compared to either physical or mental disorders alone.^50^ Within the context of our study, having more than one disability type could increase the requirement for workplace supports and may result in unique challenges to accessing workplace supports. Qualitative research is required to unpack the findings from our study to build an understanding of how living with more than one disability contributes to unmet workplace support needs.

In our sample of nearly equal proportions of men and women, we highlighted sex/gender differences in the likelihood of reporting unmet workplace support needs. Findings align with hypothesis two and indicated that study participants who were women, regardless of disability, were more likely to report unmet workplace support needs when compared to their counterparts who were men. Our findings can be explained by a body of previous research which also show that both sex (e.g., prevelance of certain disabling health conditions, symptom severity, and functional limitations) and gender differences (e.g., elder care responsibilities, parenting, work experiences) can determine physical and psychosocial exposures in the work environment and their impact on person‐job fit.^30^, ^31^, ^32^, ^33^, ^34^, ^35^, ^36^, ^38^ Drawing from previous research, both sex‐ and gender‐based factors may also determine need and use of workplace supports. Of note, our study used a one‐item open‐ended self‐report question to capture sex and gender dimensions. Accordingly, we were unable to tease apart the specific biological or sociocultural differences between men and women that may inform the availability and utilization of workplace supports.^3^, ^33^, ^35^, ^38^ Additional survey research using specific measures for sex and gender could be beneficial to advance the application of a sex/gender‐based analytical approach to understand unmet workplace support needs.^51^ Nonetheless, our findings point to the importance of considering sex and gender in the design and delivery of programs to address unmet workplace support needs.

Results indicate the intersection between disability status and type and sex/gender. In support of hypothesis three, we found that the likelihood of unmet workplace support needs was greatest for participants who were women living with a disability. The likelihood of unmet workplace support needs was elevated for women living with both a physical and nonphysical disability.^24^, ^39^ Disability and sex/gender represent individual identities that each can individually determine access to job accommodations and benefits within the workplace. When taken together, they represent overlapping identities that exacerbate the likelihood of unmet workplace support needs.^18^, ^20^, ^24^ Through an intersectional lens, each identity is informed by intersecting structures of power that can emerge from macrolevel factors (e.g., ableism, sexism) that can contribute to the disadvantage that women with disabilities may face to having their workplace support needs met.^20^, ^22^, ^23^, ^24^ Although our research shows that the types of workplace supports required may be similar for participants with and without disabilities, the extent to which they are used may differ according to individual identities. Being a woman and having one or more disabilities can contribute to intersecting disadvantages related to working experiences or employment conditions. For instance, previous studies highlight the specific challenges faced by women and people with disabilities, including difficulties communicating needs, receiving less assistance, and negative reactions from others in response to requests for help.^12^, ^52^, ^53^, ^54^ Drawing from our findings, a uniform approach to delivering workplace support to employees with and without disabilities within the workplace may not be appropriate. Instead, targeted strategies that foster utilization of workplace supports that account for sex/gender and disability should be considered. Moving forward, intersectional research approaches should continue to be used to examine how different personal characteristics (e.g., race, class, sexuality) can contribute to inequities within the workplace for people with and without disabilities.

Our study has both study strengths and limitations. First, we have recruited a diverse sample that enabled us to compare unmet workplace support needs according to disability and sex/gender differences. Additional research using a representative sample reflecting a range of job sectors and employment contexts can enable us to advance findings and produce recommendations that may be generalizable to the broader working population. We apply an intersectional framework to determine how disability and sex/gender may overlap and impact unmet workplace support needs. Our study procedures provide nuance to our understanding of inequities related to the need and use of job accommodations, work modifications, and health benefits for people with and without disabilities.^24^ Our quantitative application of intersectionality is limited in its ability to construct meaning in the different overlapping identities that contribute to unmet workplace support needs.^24^ Also, our study is limited to the number of intersecting identities that were examined. Future research using qualitative methodologies can enable a deeper understanding of how a greater number of intersecting identities can contribute to unmet workplace support needs.^22^, ^23^ Lastly, our sample consisted of participants living with physical and nonphysical disabilities. Due to sample size limitations, we were not able to examine how different nonphysical disabilities contribute to the availability and use of workplace supports. Future studies of larger samples are needed to examine the relationship between specific disabling health conditions and the likelihood of unmet workplace support needs.

Promoting the provision and use of workplace supports represents an important strategy to address work limitations for people with and without disabilities. Yet, for women and people living with both a physical and nonphysical disability, workplace support needs are more likely to be unmet. Through the lens of intersectionality, the overlap between disability and sex/gender can exacerbate unmet workplace support needs. Findings point to variability in the working experiences of people living with disabilities and highlight the need for additional research to expand on the intersection between personal and disability factors that shape working conditions. Above all, our research underscores the importance of policies and programs that are available to workers with and without disabilities to address unmet workplace support needs and promote sustained employment participation.

## CONFLICTS OF INTEREST

The authors declare that there are no conflicts of interest.

## DISCLOSURE BY AJIM EDITOR OF RECORD

Paul A. Landsbergis declares that he has no conflict of interest in the review and publication decision regarding this article.

## AUTHOR CONTRIBUTIONS

*Study conceptualization*: Arif Jetha, Monique A. M. Gignac, and Kathleen A. Martin Ginis. *Data collection*: Arif Jetha, Monique A. M. Gignac, and Kathleen A. Martin Ginis. *Data analysis*: Arif Jetha and Selahadin Ibrahim. *Manuscript preparation*: Arif Jetha, Monique A. M. Gignac, Selahadin Ibrahim, and Kathleen A. Martin Ginis.

## ETHICS APPROVAL AND INFORMED CONSENT

Study protocol was reviewed by the University of Toronto Research Ethics Board (REB# 36184). All participants provided informed consent before survey completion.

## Supporting information

Supporting information.

## Data Availability

Aligning with the requirements of our research ethics protocol, data are not shared.

## APPENDIX A

See: Table A1

**Table A1:** Multivariable logistic regression model examining unmet workplace support needs when considering the interaction between sex/gender and disability type (*n* = 1796)

	**OR**	**95% CI**
Age (years)		
18–35	Ref	
36–50	**0.70**	**0.54, 0.91**
>50	0.84	0.63, 1.10
Marital status		
Married/living as married	Ref	
Widowed/divorced/separated	0.92	0.72, 1.16
Never married	1.10	0.79, 1.53
Education level		
Primary to high school	1.05	0.79, 1.39
Some postsecondary	Ref	
Graduated postsecondary	0.95	0.75, 1.22
Pain (0–10)	1.01	0.96, 1.08
Fatigue (0–10)	0.99	0.94, 1.1
Self‐rated health (1–5)	**0.84**	**0.74, 0.94**
Productivity loss (WLQ: 0–28.6)	**0.96**	**0.93. 0.99**
Work hours (h/week)	0.99	0.98, 1.00
Perceived job stress (1–5)	1.11	0.99, 1.26
Perceived job control (1–5)	1.05	0.96, 1.14
Absenteeism (0–90)	1.00	0.99, 1.01
Primary independent variables and interaction effects		
Men, no disability	Ref	
Men, physical disability	1.57	0.97, 2.53
Men, nonphysical disability	**2.27**	**1.24, 4.15**
Men, mental and nonphysical disability	**1.64**	**1.09, 2.49**
Women, no disability	**1.54**	**1.13, 2.09**
Women, physical disability	**2.33**	**1.44, 3.78**
Women, nonphysical disability	**2.38**	**1.46, 3.88**
Women, mental and nonphysical disability	**2.73**	**1.83, 4.08**

*Note*: Bolded estimates represent those that are significantly related to unmet workplace support needs; Hosmer and Lemeshow goodness of fit test (*χ* ^2^ = 5.47(8), *p* = .71).

Abbreviations: OR, odds ratio; Ref, reference category; WLQ, work limitations questionnaire.
