# Supplementary material for: Disability and sex/gender intersections in unmet workplace support needs: Findings from a large Canadian survey of workers
Source: Am J Ind Med. 2020 Nov 24;64(2):149–61. doi: 10.1002/ajim.23203 (PMC7839541; doi:10.1002/ajim.23203)
Supplement: Supplementary file 1 — Supporting information. [file AJIM-64-149-s001.docx]

**SUPPLEMENT**

**Table 1.** Multivariable logistic regression model examining unmet workplace support needs when considering the interaction between sex/gender and disability type (n = 1,796).

|  | **OR** | **95%CI** |
| --- | --- | --- |
| Age group |  |  |
| 18-35 years | Ref |  |
| 36-50 years | **0.70** | **0.54, 0.91** |
| >50 years | 0.84 | 0.63, 1.10 |
| Marital Status |  |  |
| Married/living as married | Ref |  |
| Widowed/divorced/separated | 0.92 | 0.72, 1.16 |
| Never married | 1.10 | 0.79, 1.53 |
| Education level |  |  |
| Primary to high school | 1.05 | 0.79, 1.39 |
| Some post-secondary | Ref |  |
| Graduated post-secondary | 0.95 | 0.75, 1.22 |
| Pain (0-10) | 1.01 | 0.96, 1.08 |
| Fatigue (0-10) | 0.99 | 0.94, 1.1 |
| Self-rated health (1-5) | **0.84** | **0.74, 0.94** |
| Productivity loss (WLQ: 0-28.6) | **0.96** | **0.93. 0.99** |
| Work hours | 0.99 | 0.98, 1.00 |
| Perceived job stress (1-5) | 1.11 | 0.99, 1.26 |
| Perceived job control (1-5) | 1.05 | 0.96, 1.14 |
| Absenteeism (0-90) | 1.00 | 0.99, 1.01 |
| **Primary independent variables and interaction effects** | | |
| Men, no disability | ref |  |
| Men, physical disability | 1.57 | 0.97, 2.53 |
| Men, mental disability | **2.27** | **1.24, 4.15** |
| Men, mental and physical disability | **1.64** | **1.09, 2.49** |
| Women, no disability | **1.54** | **1.13, 2.09** |
| Women, physical disability | **2.33** | **1.44, 3.78** |
| Women, mental disability | **2.38** | **1.46, 3.88** |
| Women, mental and physical disability | **2.73** | **1.83, 4.08** |
| **Notes:** OR = odds ratio; WLQ = work limitations questionnaire, bolded estimates represent those that are significantly related to unmet workplace support needs; ref = reference category; Hosmer and Lemeshow goodness of fit test (X^2^= 5.47(8), p=0.71). | | |
